# DNAM-1-Stimulated NK-92 Cells Exert Preferential Cytotoxic and Apoptosis-Associated Effects on Hormone-Independent Solid Tumor Cell Lines

**DOI:** 10.3390/ijms27177588

**Published:** 2026-08-25

**Authors:** Mohammadreza Dastouri, Fatima Elmusa

**Affiliations:** 1 Department of Medical Biology, School of Medicine, Ankara Medipol University, Ankara 06570, Türkiye; 2 Department of Molecular Biology, Institution of Graduate Schools, Eskisehir Technical University, Eskisehir 26470, Türkiye; fatima.almousa1998@gmail.com

**Keywords:** NK-92 cells, DNAM-1, CD226, hormone-independent solid tumors, prostate cancer, neuroblastoma, apoptosis, intrinsic pathway, extrinsic pathway, immunofluorescence

## Abstract

Anti-CD226 antibody-mediated stimulation of NK-92 cells (sNK-92) represents a potential immunotherapeutic approach; however, its cytotoxic and apoptosis-associated effects in hormone-independent solid tumors remain insufficiently characterized. This study investigated the activity of sNK-92 cells against PC3 castration-resistant prostate cancer and SH-SY5Y neuroblastoma cell lines, using PNT1A normal prostate epithelial and BJ normal dermal fibroblast cells as non-malignant controls. Cytotoxicity was assessed by CCK-8 assay at target-to-effector (T:E) ratios of 1:1, 1:5, and 1:10, and markers historically associated with the intrinsic (BAX, caspase-9), extrinsic (caspase-8), and executioner (caspase-3) apoptotic pathways were evaluated quantitatively by ImageJ-based corrected total cell fluorescence (CTCF) analysis. sNK-92 cells produced significant ratio-dependent cytotoxicity against PC3 and SH-SY5Y cells, reaching 23.76% and 26.29%, respectively, at the 1:10 T:E ratio, with sNK-92 producing significantly greater cytotoxicity than unstimulated NK-92 at this ratio in both cell lines and additionally at the 1:5 ratio in SH-SY5Y cells; no significant reduction in CCK-8 viability was detected in PNT1A or BJ cells at any ratio. Quantitative immunofluorescence analysis demonstrated substantially increased relative fluorescence intensity of all four apoptosis-associated markers in sNK-92-treated PC3 and SH-SY5Y cells compared with their corresponding controls and, in most comparisons, with NK-92-treated cells, whereas changes observed in the PNT1A and BJ non-malignant models examined were markedly smaller. These findings provide preliminary quantitative evidence that anti-CD226-stimulated NK-92 cells exert a preferential cytotoxic effect on the tumor cell models examined, relative to the non-malignant models tested, and induce changes in apoptosis-associated markers consistent with engagement of apoptotic signaling. Further orthogonal validation is warranted.

## 1. Introduction

Hormone-independent solid tumors, including castration-resistant prostate cancer (CRPC) and high-risk neuroblastoma, pose a formidable clinical challenge owing to their intrinsic resistance to conventional treatment modalities and the consequent scarcity of effective therapeutic options. Prostate cancer ranks among the most frequently diagnosed malignancies in men globally, with an estimated 1.4 million new cases reported in 2020 alone [1,2]. Its CRPC form—defined by disease progression despite androgen deprivation therapy—is associated with markedly diminished survival outcomes, resistance to standard chemotherapy, and substantial therapeutic limitations [3]. Neuroblastoma, a malignancy arising from sympathoadrenal progenitor cells of the neural crest, represents the most common extracranial solid tumor of childhood and accounts for approximately 15% of all pediatric cancer-related deaths [4,5]. High-risk subtypes exhibit a propensity for metastatic dissemination and demonstrate multidrug resistance, resulting in unsatisfactory long-term prognosis despite aggressive multimodal treatment [5], motivating growing interest in cellular immunotherapy approaches for pediatric and young adult solid tumors [6]. The biological aggressiveness and hormone-independent character shared by these tumors underscore the urgent need for novel, mechanism-based therapeutic interventions.

Cancer immunotherapy has emerged as a transformative paradigm in oncology, offering the prospect of durable antitumor responses through the selective mobilization of immune effector mechanisms against malignant cells [7,8], although NK-cell-based approaches continue to face distinct challenges in solid tumors that are the focus of ongoing preclinical and clinical development [9]. Among the cellular components of the immune system, natural killer (NK) cells occupy a central position in innate antitumor surveillance. As cytotoxic lymphocytes, NK cells are capable of eliminating transformed cells without prior antigen sensitization, operating through a tightly regulated balance of activating and inhibitory surface receptors [10,11]. Upon engagement of stress-induced ligands on tumor cells, NK cells deploy multiple effector mechanisms: the polarized secretion of lytic granules containing perforin and granzymes, the engagement of death receptor pathways via FasL and TRAIL, and the release of immunomodulatory cytokines including interferon-γ (IFN-γ) and tumor necrosis factor-α (TNF-α) [12]. These mechanisms converge on the activation of apoptotic cascades within target cells through both the intrinsic (mitochondrial) and extrinsic (death receptor-mediated) pathways.

The intrinsic apoptotic pathway is principally governed by the Bcl-2 family of proteins, with pro-apoptotic BAX promoting mitochondrial outer membrane permeabilization and cytochrome c release, leading to caspase-9 activation and subsequent executioner caspase engagement [13,14]. Conversely, the extrinsic pathway is initiated by the binding of death ligands to cognate death receptors, resulting in the assembly of the death-inducing signaling complex (DISC) and the activation of caspase-8 [14,15]. Both pathways ultimately converge on caspase-3, the principal executioner caspase responsible for the proteolytic dismantling of cellular integrity and the morphological hallmarks of apoptosis [14].

DNAM-1 (also known as CD226, DNAX Accessory Molecule-1) is a transmembrane activating receptor expressed on NK cells that plays a pivotal role in immune synapse formation and the initiation of cytolytic responses [16]. It should be noted that DNAM-1 and CD226 refer to the same receptor; the term CD226 is retained herein when referring specifically to the agonistic antibody (anti-CD226 clone NewE1) employed for NK-92 cell stimulation. As an activating receptor, DNAM-1 recognizes its cognate ligands CD155 (PVR) and CD112 (nectin-2), which are frequently overexpressed on the surface of various tumor cells, thereby providing a molecular basis for selective NK-mediated tumor recognition [17,18]. Agonistic antibodies directed against DNAM-1 have demonstrated the capacity to potentiate NK cell effector functions, positioning this receptor as an attractive target for antibody-based NK cell activation strategies [19].

NK-92 is an interleukin-2-dependent NK cell line derived from a patient with non-Hodgkin lymphoma, which exhibits broad-spectrum natural cytotoxicity against diverse tumor cell types and has demonstrated a favorable safety profile in early-phase clinical trials, supporting its continued development as an off-the-shelf cellular immunotherapy platform [20,21,22,23,24,25]. Our research group has previously demonstrated that stimulation of NK-92 cells with agonistic antibodies targeting activating receptors significantly augments their cytotoxic and apoptotic effects against cancer cells while exerting no appreciable cytotoxicity against normal cells, as demonstrated using anti-CD226 (clone NewE1) in triple-negative breast cancer and anti-KIR2DL4 in HER2+/HER2− breast cancer models [26,27]. Enhancement of NK cytotoxicity mediated by DNAM-1 has also been reported in sarcoma models, further supporting the therapeutic potential of this approach [28].

PC3 and SH-SY5Y were selected as tumor models in which DNAM-1 stimulation is likely to be particularly relevant. Notably, PC3 and related castration-resistant prostate cancer cell lines have been reported to express high surface levels of the DNAM-1 ligands CD155 (PVR) and Nectin-2 (CD112), with DNAM-1 identified as one of the key NK-cell receptors involved in the recognition of prostate tumor cells [29]. Similarly, DNAM-1 engagement with CD155 has been shown to play a critical role in NK-cell-mediated killing of neuroblastoma cells [30]. Despite this established biological rationale, the effect of agonistic anti-CD226 antibody-mediated NK-92 stimulation has not previously been investigated in either castration-resistant prostate cancer or neuroblastoma models, representing the specific gap addressed by the present study.

Building upon this foundational work, the present study extends the investigation of DNAM-1-stimulated NK-92 (sNK-92) cells to hormone-independent solid tumors. We evaluated the cytotoxic activity of sNK-92 cells against the PC3 CRPC and SH-SY5Y neuroblastoma cell lines using the CCK-8 viability assay across multiple target-to-effector (T:E) ratios, employing PNT1A and BJ normal cells as non-malignant controls. Changes in markers historically associated with sNK-92-induced cell death were examined through immunofluorescence (IF) analysis of BAX and caspase-9 (intrinsic pathway), caspase-8 (extrinsic pathway), and caspase-3 (executioner caspase).

## 2. Results

### 2.1. Confirmation of Anti-CD226 Antibody Binding to NK-92 Cell Membranes

**Figure 1:**
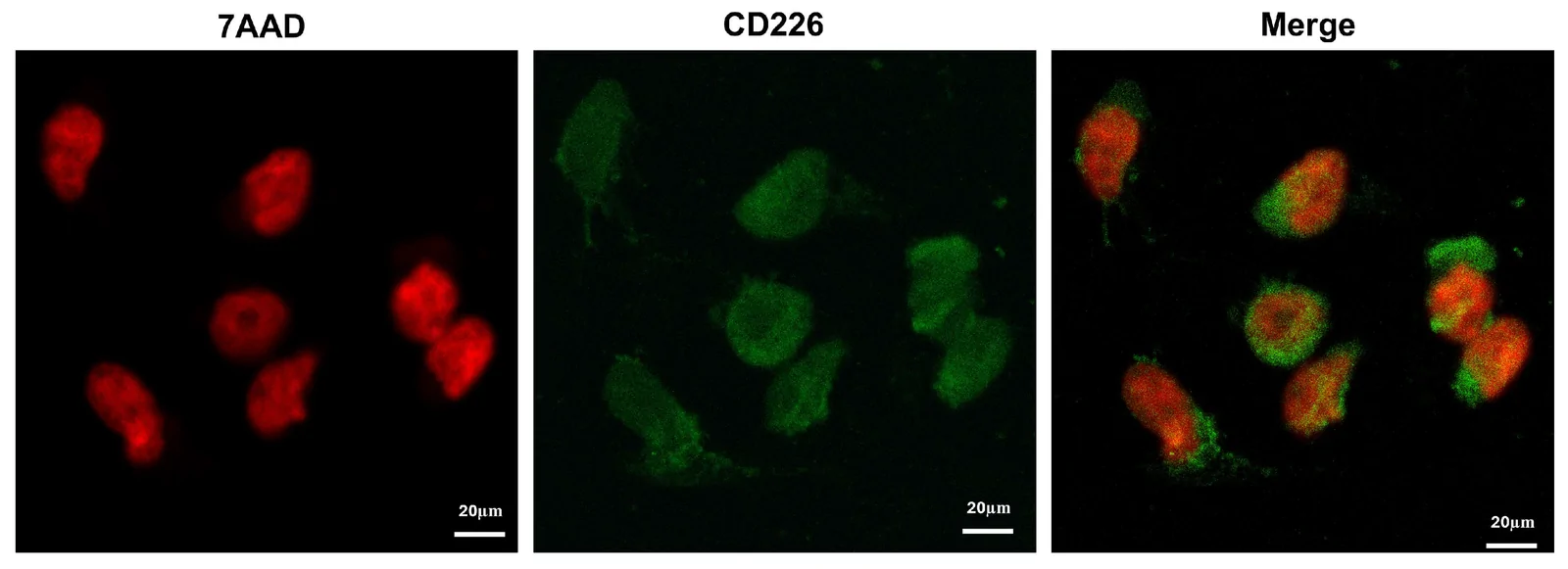
Confirmation of anti-CD226 antibody binding to the DNAM-1 receptor on NK-92 cell membranes. Cells were incubated with 1 μg/mL anti-CD226 antibody (clone NewE1) for 24 h. (**Left** panel): nuclear staining (7-AAD, red). (**Middle** panel): DNAM-1 (CD226) receptor localization (FITC, green) at the cell membrane. (**Right** panel): merged image confirming membrane-restricted antibody binding without intracellular diffusion. Images acquired by super-resolution confocal microscopy (SR-CM). Scale bar = 20 μm (applies to all panels).

Prior to co-culture experiments, the binding of the anti-CD226 agonistic antibody (clone NewE1) to the DNAM-1 receptor on NK-92 cells was verified by immunofluorescence. As shown in Figure 1, the FITC signal (green) was distinctly localized at the cell membrane surrounding the 7-AAD-stained nuclei (red) in all examined cells. The merged image confirmed complete membrane-restricted localization without intracellular diffusion, as visualized by super-resolution confocal microscopy (SR-CM), validating successful receptor engagement prior to subsequent experiments. Functional activation of NK-92 cells by this same antibody clone (NewE1) beyond receptor binding, evidenced by increased granzyme B release specifically against malignant target cells, has additionally been demonstrated in our previous work [26].

### 2.2. CCK-8 Cytotoxicity Analysis

**Figure 2:**
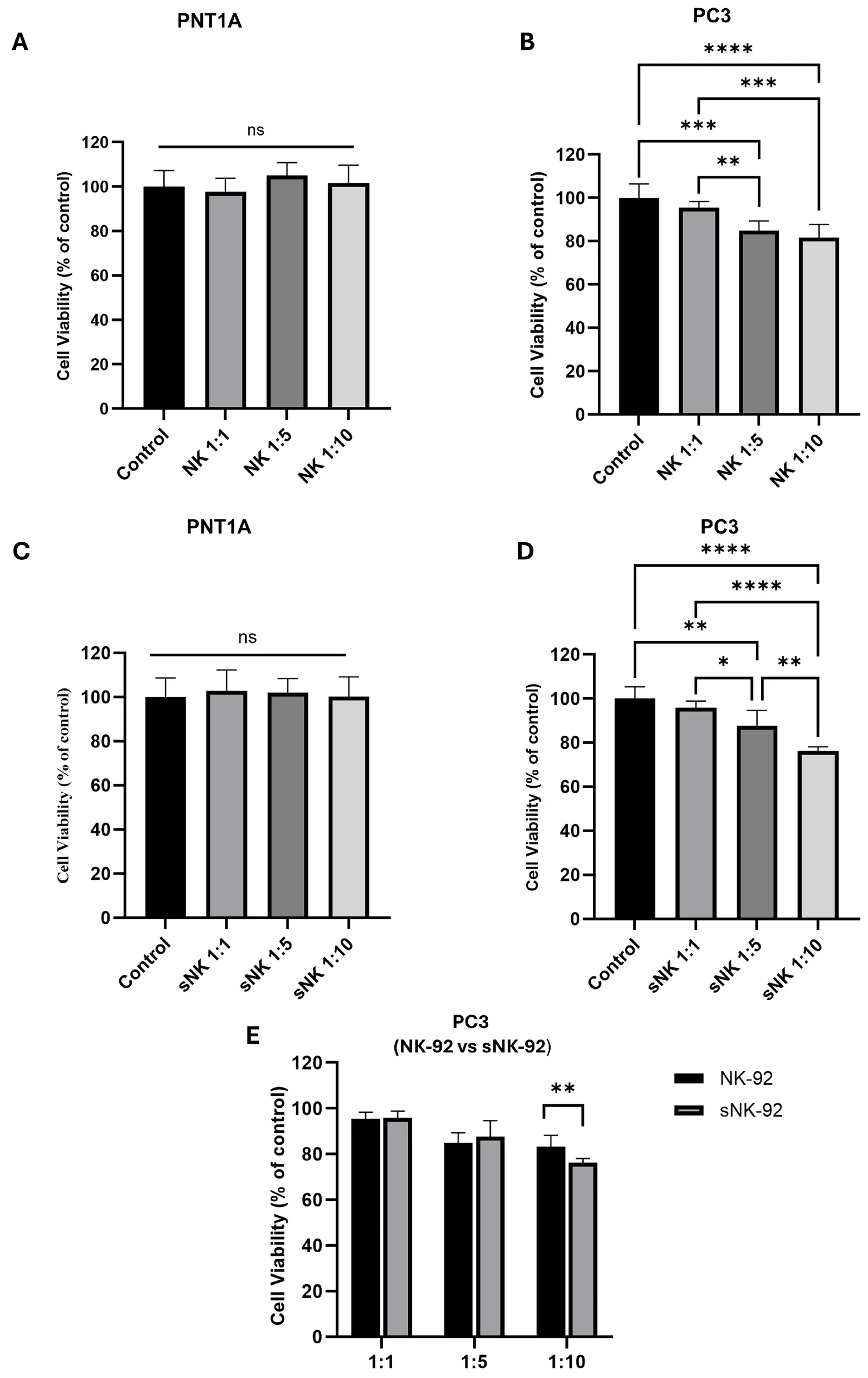
Cell viability assay (CCK-8) results of PNT1A and PC3 cells co-cultured with NK-92 and sNK-92 cells. (**A**) PNT1A normal prostate epithelial cells co-cultured with NK-92 cells. (**B**) PC3 prostate cancer cells co-cultured with NK-92 cells. (**C**) PNT1A cells co-cultured with sNK-92 cells. (**D**) PC3 cells co-cultured with sNK-92 cells. Panels (**A**–**D**) compare each treatment group with the untreated control using one-way ANOVA with Tukey’s post hoc test. (**E**) Direct comparison of cell viability (inversely reflecting cytotoxicity) between NK-92- and sNK-92-treated PC3 cells at each T:E ratio, analyzed by unpaired two-tailed Student’s *t*-test. All experiments were performed at target-to-effector (T:E) ratios of 1:1, 1:5, and 1:10. Data are presented as mean ± SD (*n* = 6 technical replicates from a single experimental preparation). * *p* < 0.05; ** *p* < 0.01; *** *p* < 0.001; **** *p* < 0.0001; ns: not significant.

**Figure 3:**
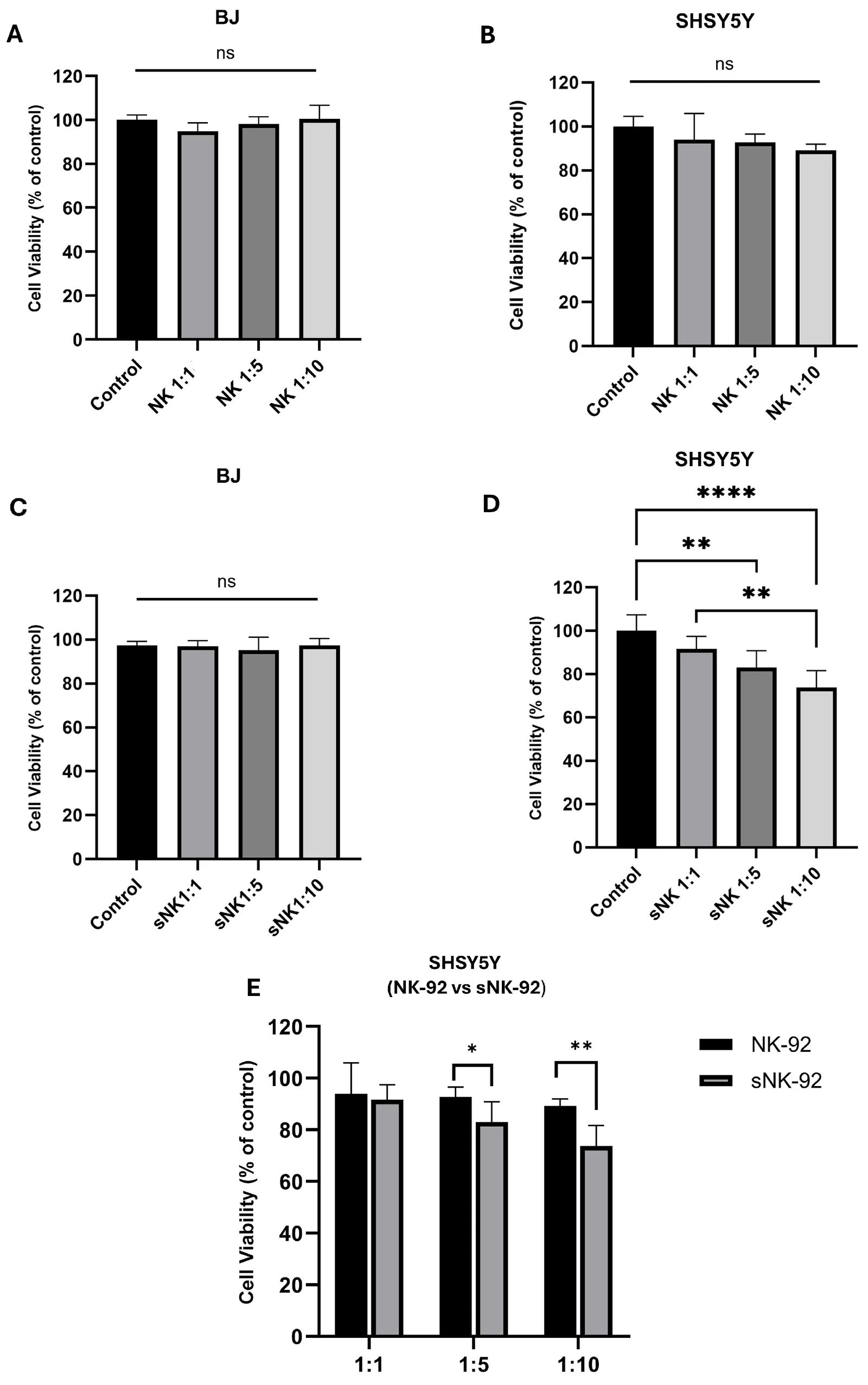
Cell viability assay (CCK-8) results of BJ and SH-SY5Y cells co-cultured with NK-92 and sNK-92 cells. (**A**) BJ normal human dermal fibroblast cells co-cultured with NK-92 cells. (**B**) SH-SY5Y neuroblastoma cells co-cultured with NK-92 cells. (**C**) BJ cells co-cultured with sNK-92 cells. (**D**) SH-SY5Y cells co-cultured with sNK-92 cells. Panels (**A**–**D**) compare each treatment group with the untreated control using one-way ANOVA with Tukey’s post hoc test. (**E**) Direct comparison of cell viability (inversely reflecting cytotoxicity) between NK-92- and sNK-92-treated SH-SY5Y cells at each T:E ratio, analyzed by unpaired two-tailed Student’s *t*-test. All experiments were performed at target-to-effector (T:E) ratios of 1:1, 1:5, and 1:10. Data are presented as mean ± SD (*n* = 6 technical replicates from a single experimental preparation). * *p* < 0.05; ** *p* < 0.01; **** *p* < 0.0001; ns: not significant.

Cell viability was assessed by CCK-8 assay following 4-h co-culture of NK-92 and sNK-92 cells with tumor (PC3, SH-SY5Y) and normal (PNT1A, BJ) cell lines. For each cell line, four experimental groups were assessed: untreated control and co-culture at target-to-effector (T:E) ratios of 1:1, 1:5, and 1:10 (*n* = 6 per group). Results are presented in Figure 2 and Figure 3.

#### 2.2.1. Cytotoxic Effects on PC3 CRPC Cells

Unstimulated NK-92 cells reduced PC3 viability in a ratio-dependent manner to 95.48 ± 2.87%, 84.91 ± 4.43%, and 81.76 ± 6.00% at T:E ratios of 1:1, 1:5, and 1:10, corresponding to cytotoxicity values of 4.52%, 15.09%, and 18.24% (Figure 2B). These reductions were statistically significant at the 1:5 and 1:10 ratios compared to control (*p* < 0.001 and *p* < 0.0001, respectively). sNK-92 cells demonstrated enhanced cytotoxicity against PC3 cells, reducing viability to 95.86 ± 2.97%, 87.65 ± 7.04%, and 76.24 ± 1.90% (cytotoxicity: 4.14%, 12.35%, 23.76%; Figure 2D). Compared to control, cytotoxicity reached statistical significance at the 1:5 (*p* < 0.05) and 1:10 (*p* < 0.0001) ratios, while the 1:1 ratio did not reach significance (*p* > 0.05). A statistically significant difference was also observed between the 1:5 and 1:10 ratio groups (*p* < 0.01), confirming a progressive, ratio-dependent pattern of viability reduction. Notably, direct comparison of NK-92 and sNK-92 cytotoxicity at each T:E ratio (Figure 2E) confirmed that sNK-92 cells produced significantly greater cytotoxicity than NK-92 cells at the 1:10 T:E ratio (*p* < 0.01), with no significant difference at the 1:1 or 1:5 ratios, reflecting the dose-dependent enhancement conferred by CD226 receptor stimulation at higher effector concentrations.

#### 2.2.2. Cytotoxic Effects on SH-SY5Y Neuroblastoma Cells

Unstimulated NK-92 cells did not significantly affect SH-SY5Y viability at any T:E ratio (94.00 ± 11.99%, 92.70 ± 3.84%, 89.29 ± 2.68%; *p* > 0.05 for all; Figure 3B). In marked contrast, sNK-92 cells elicited a significant ratio-dependent cytotoxic response, reducing viability to 91.64 ± 5.82%, 83.03 ± 7.85%, and 73.71 ± 7.89% (cytotoxicity: 8.36%, 16.97%, 26.29%; Figure 3D). Compared to control, cytotoxicity reached statistical significance at the 1:5 (*p* < 0.01) and 1:10 (*p* < 0.0001) ratios, while the 1:1 ratio did not reach significance (*p* > 0.05). A statistically significant difference was observed between the 1:1 and 1:10 T:E ratio groups (*p* < 0.01), further confirming the ratio-dependent nature of sNK-92 cytotoxicity against SH-SY5Y cells. Direct comparison of NK-92 and sNK-92 cytotoxicity at each T:E ratio (Figure 3E) confirmed that sNK-92 cells produced significantly greater cytotoxicity than NK-92 cells at both the 1:5 (*p* < 0.05) and 1:10 (*p* < 0.01) T:E ratios, with no significant difference at the 1:1 ratio. This contrast between the modest activity of unstimulated NK-92 cells and the robust sNK-92 response underscores the critical role of CD226 receptor stimulation in enabling effective targeting of SH-SY5Y neuroblastoma cells.

#### 2.2.3. Absence of Cytotoxicity in Normal Cell Lines

Neither NK-92 nor sNK-92 cells induced statistically significant changes in viability of PNT1A or BJ cells at any T:E ratio (Figure 2A,C and Figure 3A,C; *p* > 0.05 for all). Viability of PNT1A cells remained at 97.62–105.08% under NK-92 and 100.20–102.85% under sNK-92 co-culture. Viability of BJ cells was equally unaffected (94.81–100.41% for NK-92; 97.70–100.06% for sNK-92), consistent with a preferential cytotoxic effect of sNK-92 cells on the tumor models examined, relative to the PNT1A and BJ non-malignant models tested.

### 2.3. Immunofluorescence Analysis of Apoptosis-Associated Markers

**Figure 4:**
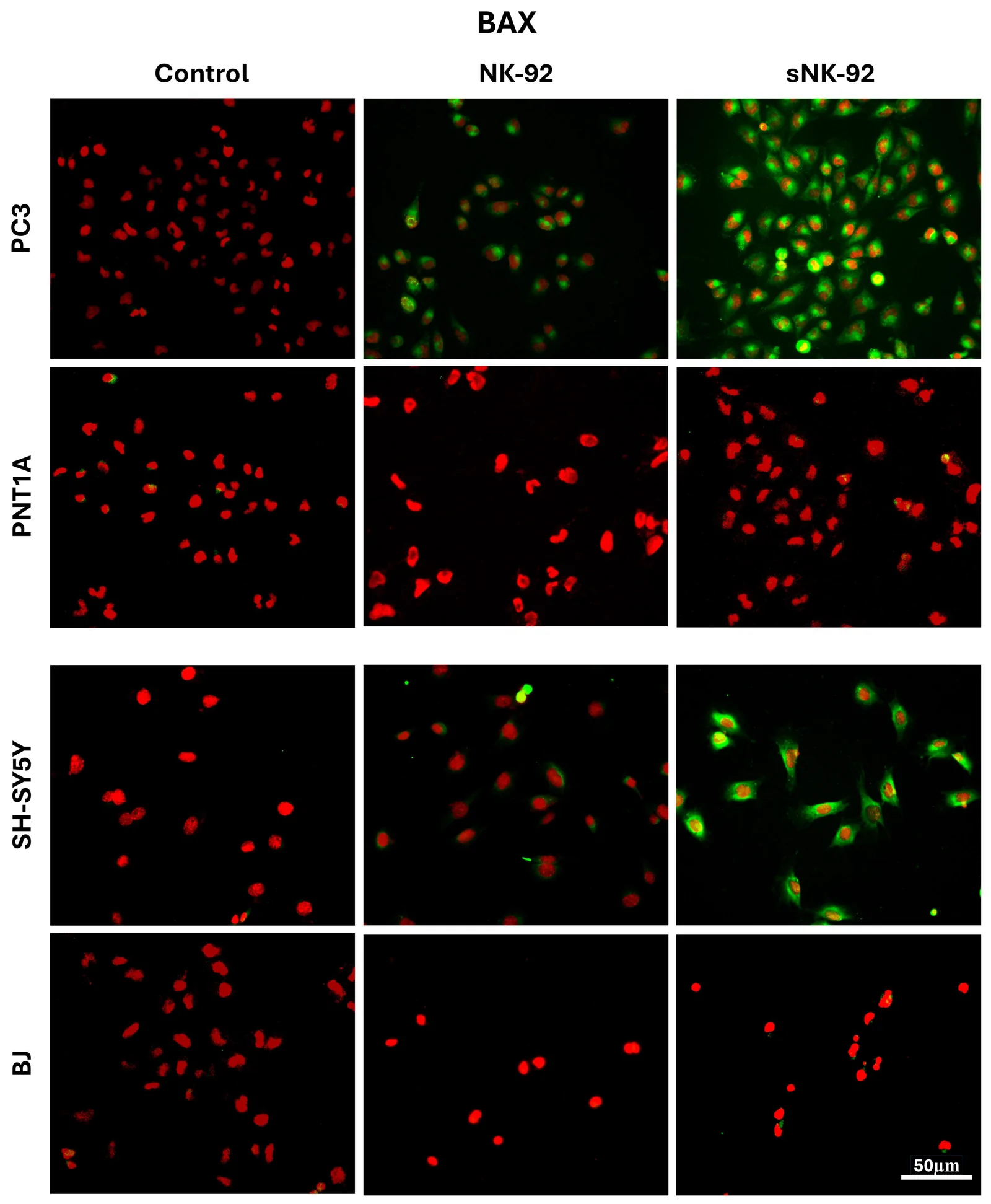
Immunofluorescence analysis of BAX protein expression in PC3, PNT1A, SH-SY5Y, and BJ cells co-cultured with NK-92 and sNK-92 cells. Green signal (FITC): BAX protein localization; red signal (7-AAD): nuclei. Representative merged fluorescence images showing typical staining patterns are presented. Images acquired using a Zeiss Axio Observer fluorescence microscope (Carl Zeiss AG, Oberkochen, Germany) at 40× magnification. Scale bar = 50 μm (applies to all panels).

**Figure 5:**
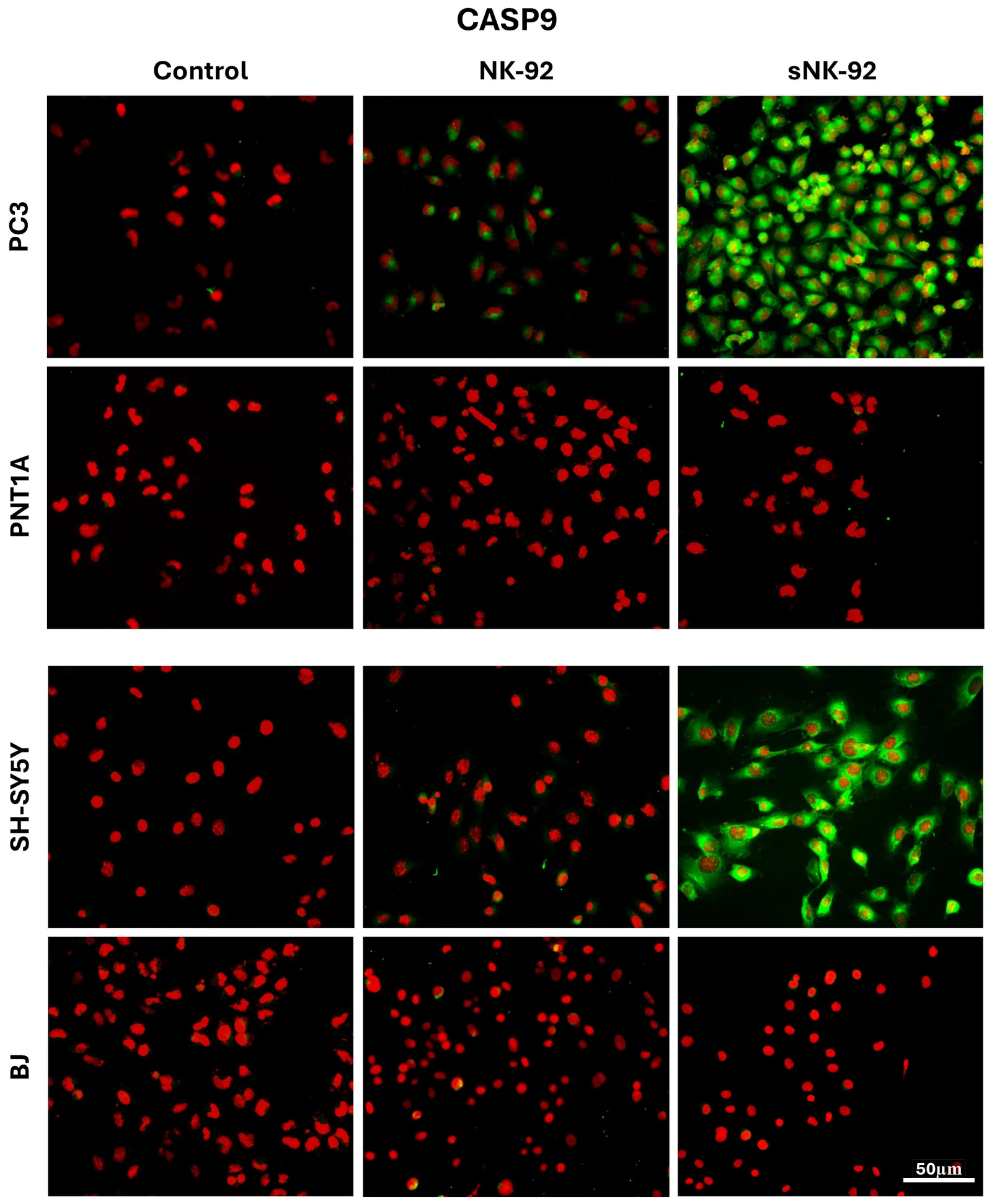
Immunofluorescence analysis of caspase-9 (CASP9) protein expression in PC3, PNT1A, SH-SY5Y, and BJ cells co-cultured with NK-92 and sNK-92 cells. Green signal (FITC): CASP9 protein localization; red signal (7-AAD): nuclei. Representative merged fluorescence images showing typical staining patterns are presented. Images acquired using a Zeiss Axio Observer fluorescence microscope (Carl Zeiss AG, Oberkochen, Germany) at 40× magnification. Scale bar = 50 μm (applies to all panels).

**Figure 6:**
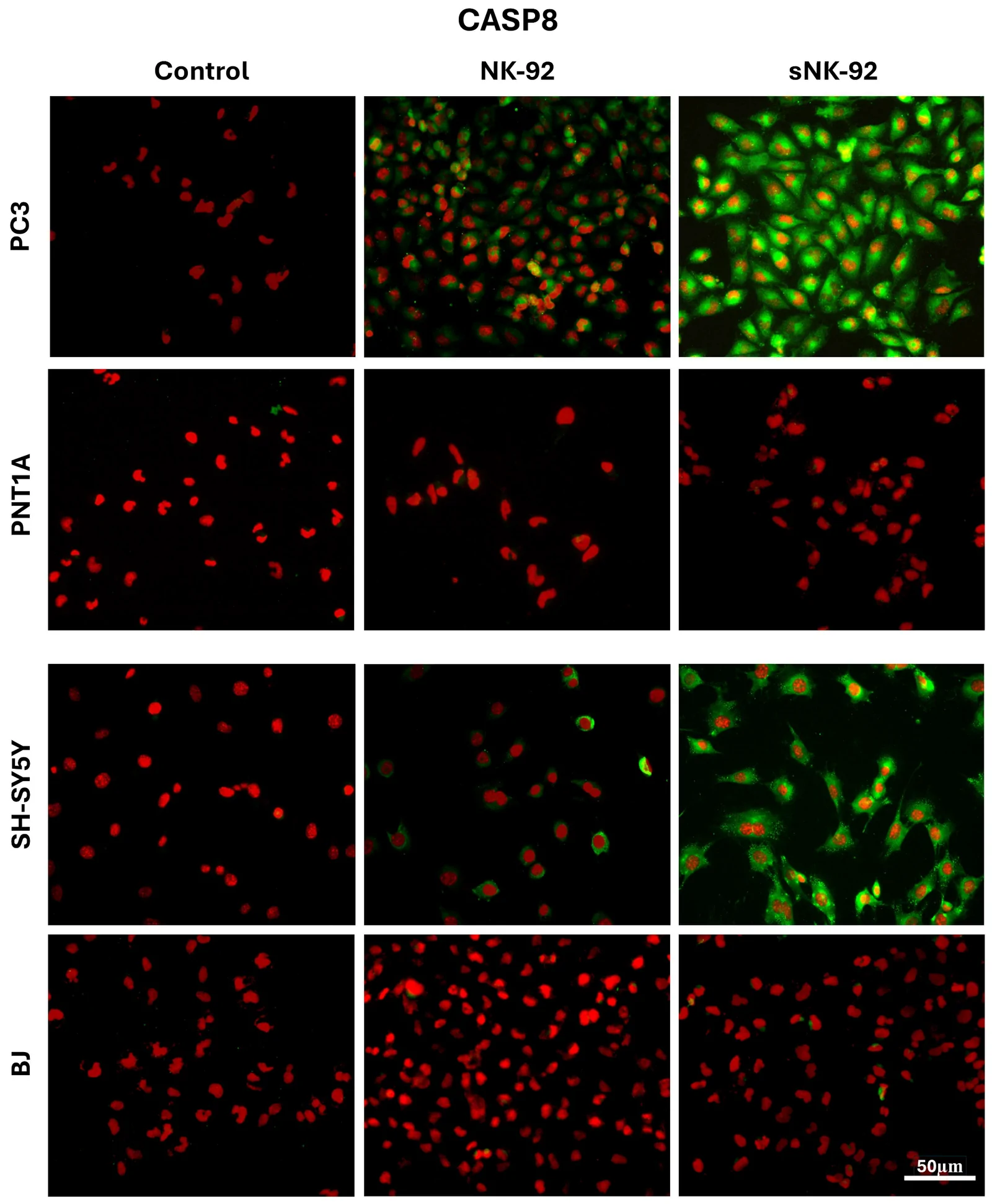
Immunofluorescence analysis of caspase-8 (CASP8) protein expression in PC3, PNT1A, SH-SY5Y, and BJ cells co-cultured with NK-92 and sNK-92 cells. Green signal (FITC): CASP8 protein localization; red signal (7-AAD): nuclei. Representative merged fluorescence images showing typical staining patterns are presented. Images acquired using a Zeiss Axio Observer fluorescence microscope (Carl Zeiss AG, Oberkochen, Germany) at 40× magnification. Scale bar = 50 μm (applies to all panels).

**Figure 7:**
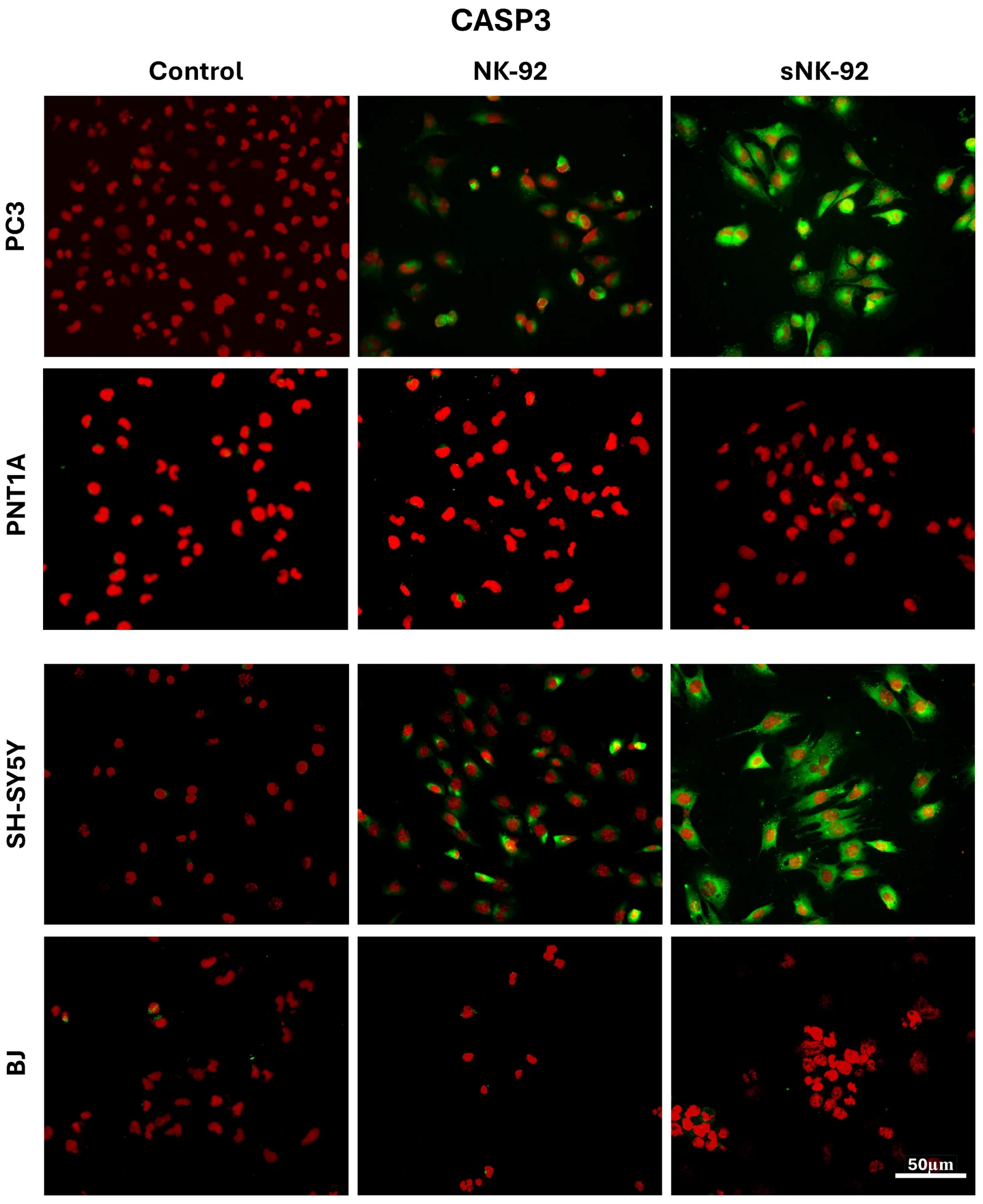
Immunofluorescence analysis of caspase-3 (CASP3) protein expression in PC3, PNT1A, SH-SY5Y, and BJ cells co-cultured with NK-92 and sNK-92 cells. Green signal (FITC): CASP3 protein localization; red signal (7-AAD): nuclei. Representative merged fluorescence images showing typical staining patterns are presented. Images acquired using a Zeiss Axio Observer fluorescence microscope (Carl Zeiss AG, Oberkochen, Germany) at 40× magnification. Scale bar = 50 μm (applies to all panels).

**Figure 8:**
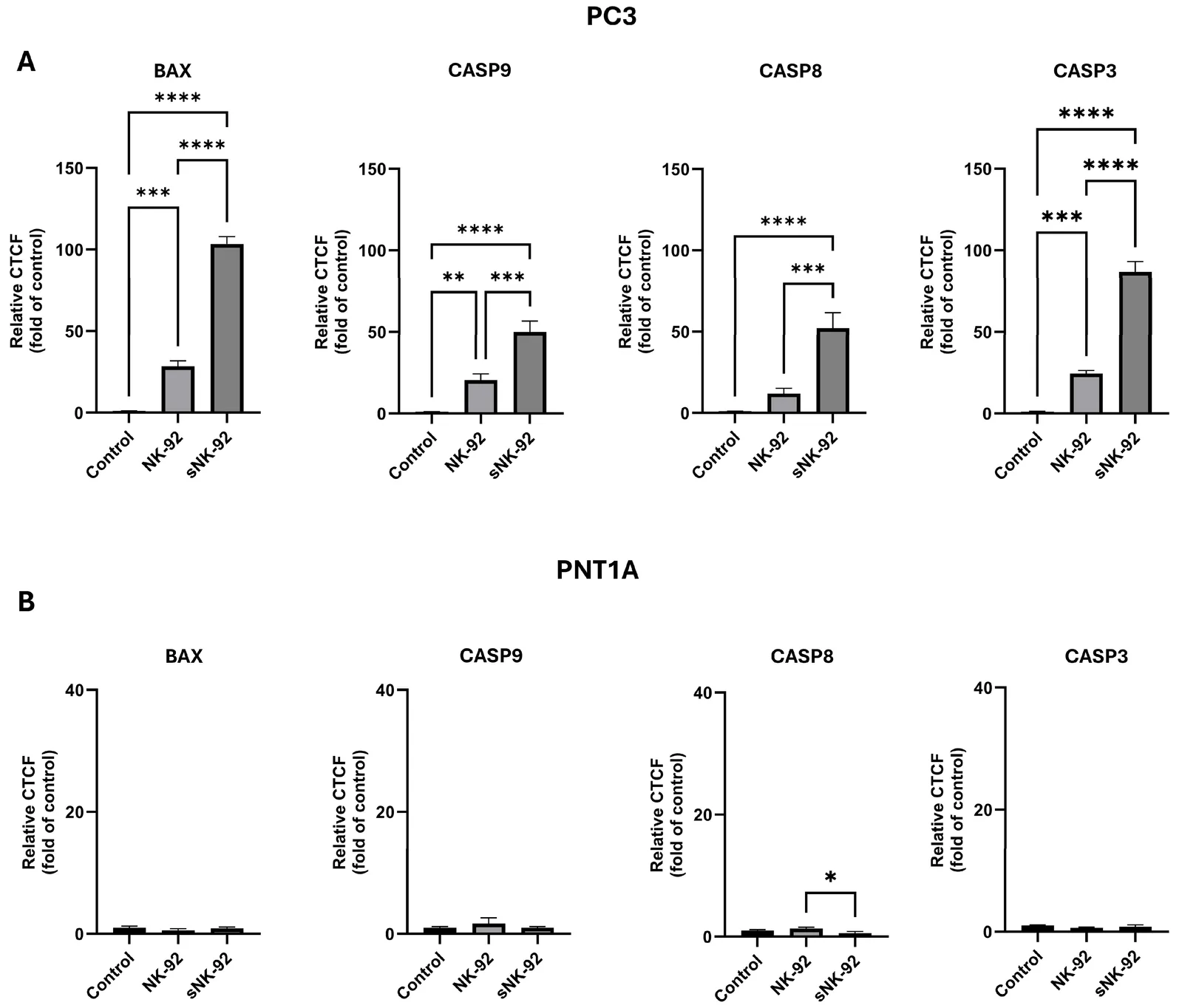
Quantitative analysis of apoptosis-associated protein fluorescence in PC3 and PNT1A cells. Relative corrected total cell fluorescence (CTCF) values for BAX, CASP9, CASP8, and CASP3 are shown for the control, NK-92, and sNK-92 groups in (**A**) PC3 and (**B**) PNT1A cells. For each condition, individual cellular CTCF measurements were averaged within each image, and three image-level values were analyzed per group (*n* = 3 image fields). Data were normalized to the mean of the corresponding control group and are presented as fold of control (mean ± SD). Statistical comparisons were performed using ordinary one-way ANOVA followed by Tukey’s multiple-comparisons test. Only statistically significant pairwise comparisons are indicated by brackets; unmarked comparisons were not statistically significant. * *p* < 0.05, ** *p* < 0.01, *** *p* < 0.001, and **** *p* < 0.0001. Note the different *Y*-axis scales used for the PC3 and PNT1A panels.

**Figure 9:**
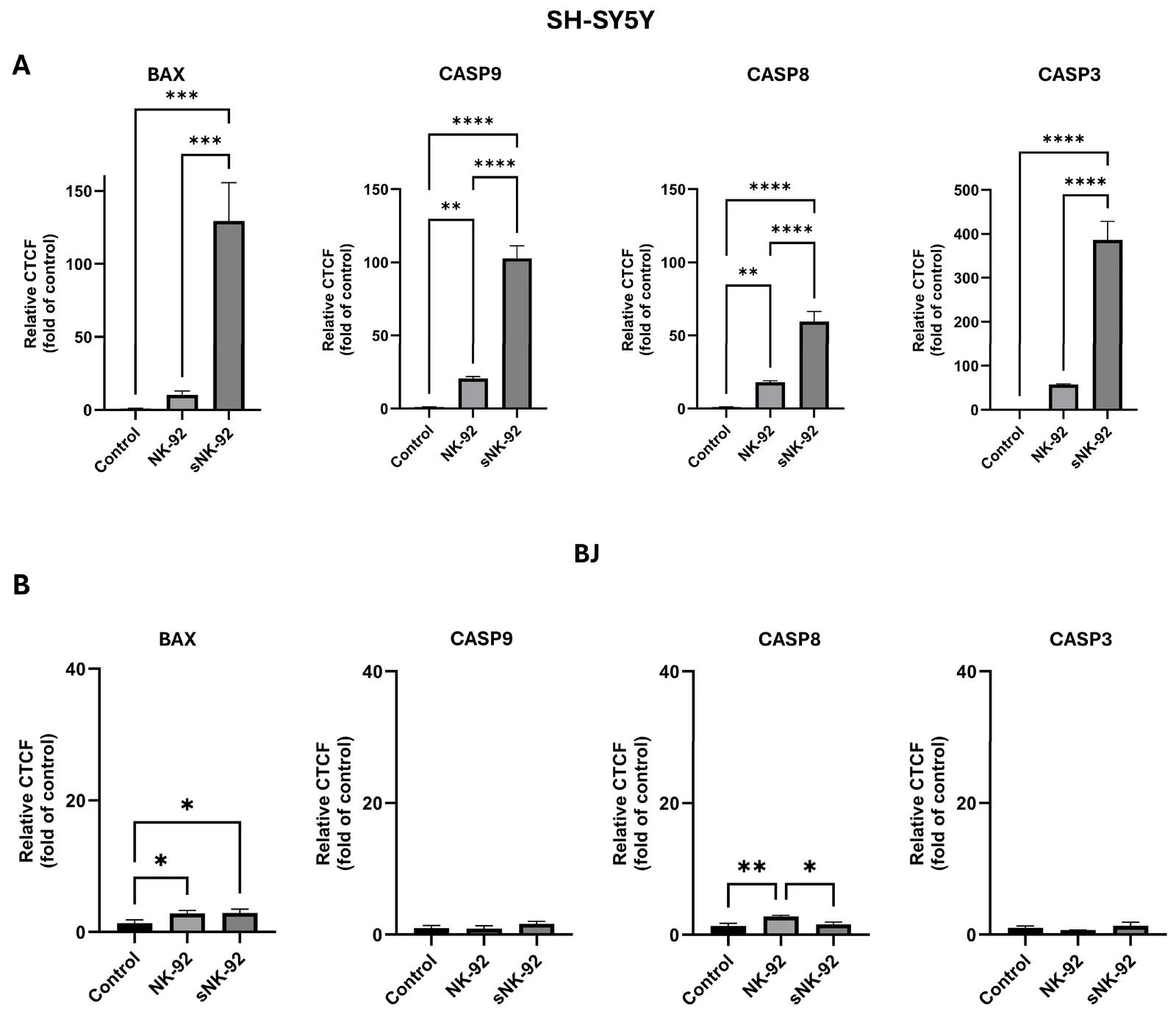
Quantitative analysis of apoptosis-associated protein fluorescence in SH-SY5Y and BJ cells. Relative corrected total cell fluorescence (CTCF) values for BAX, CASP9, CASP8, and CASP3 are shown for the control, NK-92, and sNK-92 groups in (**A**) SH-SY5Y and (**B**) BJ cells. For each condition, individual cellular CTCF measurements were averaged within each image, and three image-level values were analyzed per group (*n* = 3 image fields). Data were normalized to the mean of the corresponding control group and are presented as fold of control (mean ± SD). Statistical comparisons were performed using ordinary one-way ANOVA followed by Tukey’s multiple-comparisons test. Only statistically significant pairwise comparisons are indicated by brackets; unmarked comparisons were not statistically significant. * *p* < 0.05, ** *p* < 0.01, *** *p* < 0.001, and **** *p* < 0.0001. Note the different *Y*-axis scales used for the SH-SY5Y CASP3 panel and the remaining panels.

Immunofluorescence (IF) analysis was performed at the 1:10 T:E ratio to examine changes in the apoptosis-associated proteins BAX, CASP9, CASP8, and CASP3. The red 7-AAD signal indicates nuclear staining, whereas the green FITC signal represents the localization and relative abundance of the examined protein. Representative images are presented in Figure 4, Figure 5, Figure 6 and Figure 7 and the corresponding ImageJ-based quantitative CTCF analyses are shown in Figure 8 and Figure 9.

#### 2.3.1. Intrinsic Apoptotic Pathway: BAX and Caspase-9

The intrinsic apoptotic pathway was assessed through IF analysis of BAX and caspase-9 (CASP9) (Figure 4 and Figure 5). In the control group, no detectable green fluorescence signal was observed in any cell line. Co-culture with unstimulated NK-92 cells resulted in a modest increase in green signal, specifically in PC3 and SH-SY5Y cancer cells, while PNT1A and BJ normal cells remained signal-free. Co-culture with sNK-92 cells produced a visually greater increase in BAX and CASP9 green fluorescence in both PC3 and SH-SY5Y cells than that observed in NK-92-treated groups, consistent with the quantitative CTCF comparisons presented in Section 2.3.3. Critically, PNT1A and BJ cells co-cultured with sNK-92 showed no visually appreciable increase in BAX or CASP9 signal in these representative fields, consistent with the preferential upregulation of intrinsic apoptotic pathway markers in malignant cells confirmed quantitatively in Section 2.3.3. Representative fields showing typical staining patterns are presented.

#### 2.3.2. Extrinsic Apoptotic Pathway and Executioner Caspase: Caspase-8 and Caspase-3

Engagement of the extrinsic apoptotic pathway was evaluated through IF analysis of caspase-8 (CASP8) and caspase-3 (CASP3) (Figure 6 and Figure 7). In control cells, no green signal was detected. Co-culture with unstimulated NK-92 cells resulted in a modest CASP8 and CASP3 signal increase in PC3 and SH-SY5Y cancer cells, while PNT1A and BJ remained signal-free. sNK-92 co-culture produced a visually greater increase in CASP8 and CASP3 fluorescence in both cancer cell lines than that observed in NK-92-treated groups, consistent with the quantitative CTCF comparisons presented in Section 2.3.3. In these representative fields, PNT1A and BJ cells showed no visually appreciable signal upregulation in these representative fields under any condition. Collectively, these results indicate concurrent upregulation of markers historically associated with intrinsic (BAX, CASP9), extrinsic (CASP8), and executioner (CASP3) apoptotic pathways, preferentially in the hormone-independent solid tumor cell lines examined relative to the PNT1A and BJ non-malignant models tested; the corresponding quantitative comparisons, including the small but statistically significant changes detected in the non-malignant models, are presented in Section 2.3.3. Representative fields showing typical staining patterns are presented.

#### 2.3.3. Quantitative CTCF Analysis

To provide an objective assessment of the immunofluorescence findings, relative CTCF was calculated from three analyzed image fields per group and normalized to the corresponding control mean.

In PC3 cells, the mean relative CTCF values for the control, NK-92, and sNK-92 groups were approximately 1.00, 28.42, and 103.40 for BAX; 1.00, 20.46, and 49.94 for CASP9; 1.00, 11.98, and 52.10 for CASP8; and 1.00, 24.46, and 86.65 for CASP3, respectively (Figure 8A). The sNK-92 group exhibited significantly greater fluorescence intensity than the control and NK-92 groups for all four markers. Treatment with NK-92 also produced significant increases relative to the control for BAX, CASP9, and CASP3, whereas the Control–NK-92 comparison for CASP8 was not statistically significant.

In PNT1A cells, relative CTCF values remained low across all four markers compared with those observed in PC3 cells (Figure 8B). No significant differences were detected for BAX, CASP9, or CASP3. A small but statistically significant difference was observed between the NK-92 and sNK-92 groups for CASP8.

In SH-SY5Y cells, the mean relative CTCF values for the control, NK-92, and sNK-92 groups were approximately 1.00, 10.31, and 124.56 for BAX; 1.00, 20.56, and 102.82 for CASP9; 1.00, 18.06, and 59.49 for CASP8; and 1.00, 57.22, and 386.20 for CASP3, respectively (Figure 9A). The sNK-92 group showed significantly greater fluorescence intensity than both the control and NK-92 groups for all four markers. Treatment with NK-92 significantly increased CASP9 and CASP8 fluorescence relative to the control, whereas the corresponding comparisons for BAX and CASP3 were not significant.

In BJ cells, all mean relative CTCF values remained below approximately 3.2-fold of the corresponding control values (Figure 9B). Small but statistically significant differences were detected for BAX between the control and both NK-92-treated groups, and for CASP8 between the control and NK-92 groups and between the NK-92 and sNK-92 groups. No significant differences were observed for CASP9 or CASP3. Overall, the magnitude of the fluorescence changes in PNT1A and BJ cells was substantially lower than that observed in PC3 and SH-SY5Y tumor cells.

## 3. Discussion

The present study investigated the cytotoxic activity and apoptosis-associated marker changes in DNAM-1-stimulated NK-92 cells against PC3 CRPC and SH-SY5Y neuroblastoma cell lines, alongside PNT1A and BJ normal cells as non-malignant controls. The results demonstrate that anti-CD226 antibody-mediated stimulation significantly enhances NK-92 cytotoxic activity and apoptotic marker expression in both cancer cell lines, with substantially smaller effects observed in the PNT1A and BJ non-malignant models examined. These findings are consistent with our previous reports [26,27] and extend the therapeutic applicability of receptor-mediated NK-92 activation to a broader spectrum of hormone-independent solid malignancies.

The CCK-8 assays revealed ratio-dependent cytotoxicity with maximum values of 23.76% and 26.29% in PC3 and SH-SY5Y cells, respectively, at the 1:10 T:E ratio. Notably, direct statistical comparison confirmed that sNK-92 cells produced significantly greater cytotoxicity than NK-92 cells at the 1:10 T:E ratio in both cancer cell lines (PC3: *p* < 0.01, Figure 2E; SH-SY5Y: *p* < 0.01, Figure 3E), and additionally at the 1:5 ratio in SH-SY5Y cells (*p* < 0.05), reflecting that the enhancement conferred by CD226 stimulation becomes most apparent at higher effector concentrations. No significant cytotoxicity was detected in the PNT1A and BJ normal cell models examined under any condition, consistent with a preferential cytotoxic effect of sNK-92 activity on the tumor models tested.

The maximum cytotoxicity values recorded in this study (23.76% for PC3 and 26.29% for SH-SY5Y at the 1:10 T:E ratio) are numerically modest and warrant explanation. Several non-mutually exclusive factors likely contribute to this magnitude. First, the 4-h co-culture window, selected to align with the time point used for immunofluorescence analysis, captures only the earliest phase of NK-mediated killing. This duration also reflects a constraint common to short-term co-culture cytotoxicity assays more broadly, in which effector and target cells share a limited well volume and medium; extending the incubation period increases the risk of nutrient depletion and non-specific culture stress that could confound the interpretation of the results, rather than reflecting a deficiency specific to the present experimental design. Second, PC3 and SH-SY5Y are adherent solid-tumor cell lines that, in comparison with hematologic or suspension targets more commonly used in NK cytotoxicity assays, have been reported in the literature to display comparatively lower baseline NKG2D-ligand and DNAM-1-ligand (CD155/CD112) density and higher expression of anti-apoptotic regulators, both of which could raise the activation threshold required for efficient lysis; direct measurement of these ligands was outside the scope of the present study, which focused on antibody-mediated DNAM-1 stimulation independent of natural ligand engagement. Engagement of DNAM-1 has previously been shown to be an important contributor to NK-cell-mediated cytotoxicity against pediatric solid tumor models, including neuroblastoma-lineage cell lines [31]. Third, NK-92 is a long-term cultured cell line that, despite IL-2 supplementation, generally exhibits lower per-cell lytic efficiency than freshly isolated primary NK cells, which may further constrain the achievable cytotoxicity within a short co-culture period. Finally, the CCK-8 assay reports a population-level reduction in metabolic viability rather than a direct measure of cell lysis, and may therefore underestimate the fraction of cells that have entered early apoptotic signaling without yet showing a corresponding loss of metabolic activity; this is consistent with the comparatively larger changes captured by the CTCF-based immunofluorescence analysis of apoptosis-associated markers at the same time point (Section 2.3.3). Published work using NK-92 cells against solid tumor models has shown that increasing the effector-to-target ratio substantially increases the extent of tumor cell killing [32]; higher E:T ratios, extended co-culture durations incorporating medium-replenishment strategies, and antibody dose–response optimization represent concrete directions that could be explored in future work to increase the magnitude of the cytotoxic effect observed here.

The failure of unstimulated NK-92 cells to elicit significant cytotoxicity in SH-SY5Y cells, despite moderate activity in PC3 cells, may reflect several factors, including possible differences in the surface density of DNAM-1 ligands (CD155/CD112), which have been reported in the literature to vary across tumor types and influence the NK activation threshold [16,17]; direct measurement of ligand expression was outside the scope of the present study, which focused on antibody-mediated receptor stimulation independent of natural ligand engagement. Ligation of DNAM-1 by the anti-CD226 antibody was associated with enhanced cytotoxic activity against SH-SY5Y cells, which were otherwise less susceptible to unstimulated NK-92 lysis.

IF analysis demonstrated progressive upregulation of BAX and CASP9 in sNK-92-treated cancer cells, indicative of intrinsic mitochondria-associated apoptotic signaling. The pro-apoptotic protein BAX promotes mitochondrial outer membrane permeabilization and cytochrome c release, leading to caspase-9 activation [13], and the concurrent upregulation of both markers is consistent with our prior findings in breast cancer models [26,27]. Alongside these intrinsic pathway markers, significant CASP8 upregulation was observed in sNK-92-treated cancer cells. While caspase-8 is canonically associated with the extrinsic death receptor pathway through DISC-mediated activation downstream of FasL and TRAIL signaling [15], granzyme B secreted by activated NK cells may also directly process caspase-8 in a death receptor-independent manner [12,14]. The downstream convergence of these pathways is consistent with the elevated CASP3 expression observed in sNK-92-treated cancer cells [14].

The concurrent upregulation of four canonical apoptotic pathway markers—BAX, CASP9, CASP8, and CASP3—predominantly in cancer cells under sNK-92 conditions, with markedly smaller changes in the non-malignant models examined (Section 2.3.3), provides IF evidence consistent with differential engagement of apoptosis-associated signaling. Although direct quantification by Annexin V/PI flow cytometry was not performed, the combination of these four complementary markers spanning intrinsic, extrinsic, and executioner levels constitutes a multi-layered pattern of changes consistent with apoptosis-associated signaling [14]. None of the apoptotic markers showed visually appreciable upregulation in PNT1A or BJ normal cells by qualitative inspection, consistent with a preferential tumor selectivity that may reflect differential DNAM-1 ligand expression between tumor and normal cells reported in the literature [16,17]; as noted above, ligand expression was not directly measured in this study, and the quantitative basis for this preferential, rather than absolute, selectivity is detailed below. The present study was conducted under in vitro conditions; future studies employing in vivo tumor models, quantitative apoptosis assays, and combination strategies with immune checkpoint inhibitors will be essential to fully evaluate the translational potential of DNAM-1-stimulated NK-92 cells.

The ImageJ-based CTCF analysis provided quantitative support for the qualitative immunofluorescence observations. In both tumor cell models, sNK-92 co-culture produced pronounced increases in the relative fluorescence intensity of BAX, CASP9, CASP8, and CASP3. The magnitude of these changes was greater than that observed following co-culture with unstimulated NK-92 cells, particularly for BAX and CASP3 in SH-SY5Y cells and for BAX and CASP3 in PC3 cells. The concurrent elevation of BAX and CASP9 is consistent with changes involving the intrinsic mitochondrial apoptotic pathway, whereas increased CASP8 and CASP3 fluorescence is consistent with the involvement of extrinsic and downstream executioner components, respectively. Nevertheless, increased immunofluorescence intensity of these proteins should not be interpreted as definitive evidence of enzymatic caspase activation or complete pathway engagement.

The changes detected in the examined non-malignant cell models were substantially smaller than those observed in the tumor cells. Although several field-level comparisons reached statistical significance in PNT1A or BJ cells, their relative CTCF values generally remained close to the corresponding control levels and were markedly lower than those measured in PC3 and SH-SY5Y cells. These findings therefore support a preferential, rather than absolute, effect of sNK-92 cells on the tumor models examined. Because PNT1A and BJ represent only two non-malignant cell models, the present findings should not be generalized as evidence of universal tumor selectivity.

Quantitative CTCF analysis strengthens the objectivity of the immunofluorescence results but does not replace independent confirmation of apoptosis or protein abundance. The antibodies used in this study assessed the fluorescence of apoptosis-associated proteins and did not directly distinguish active cleaved caspases from their inactive forms. Moreover, Western blotting, Annexin V/PI flow cytometry, or another orthogonal apoptosis assay was not performed, and the IF findings should accordingly be considered preliminary and interpreted as being consistent with apoptosis-associated signaling. The quantitative image analysis was based on three image fields per group from a single experimental preparation, consistent with the technical-replicate design used throughout this study; independent biological replication represents a natural next step to confirm the generalizability of these findings. Future studies should confirm these observations using independent biological replicates, cleavage-specific protein analysis, flow-cytometric apoptosis assays, and functional assessment of DNAM-1 pathway activation.

### Study Limitations

A limitation of the present study should be acknowledged: apoptosis-associated marker changes were assessed by immunofluorescence without orthogonal confirmation by Western blotting, Annexin V/PI flow cytometry, or a functional caspase activity assay. This limitation does not undermine the internal consistency of the reported findings but constrains the strength of the mechanistic and biological conclusions that can be drawn from them.

## 4. Materials and Methods

### 4.1. Cell Lines and Culture Conditions

All cell lines were maintained at 37 °C in a humidified 5% CO_2_ atmosphere. The NK-92 cells (ATCC, Manassas, VA, USA; Cat. No. CRL-2407) were cultured in TexMACS medium (Miltenyi Biotec, Bergisch Gladbach, Germany) supplemented with 20% fetal bovine serum (FBS; Biowest, Nuaillé, France, Cat. No. S181H-500), 500 U/mL recombinant human IL-2 (PeproTech, Cranbury, NJ, USA; Cat. No. 200-02), 1% penicillin/streptomycin (Gibco, Thermo Fisher Scientific, Waltham, MA, USA; Cat. No. 10378016), and 1% L-glutamine (Gibco, Thermo Fisher Scientific, Waltham, MA, USA; Cat. No. 25030081). The PC3 (ATCC, CRL-1435), SH-SY5Y (ATCC, CRL-2266), PNT1A (ECACC, Salisbury, UK; 95012613), and BJ (ATCC, CRL-2522) cell lines were maintained in DMEM High Glucose (Sigma-Aldrich, St. Louis, MO, USA; Cat. No. D6429-500ML) supplemented with 10% FBS, 1% penicillin/streptomycin, and 1% L-glutamine.

### 4.2. Stimulation of NK-92 Cells with Anti-CD226 Monoclonal Antibody Clone NewE1

NK-92 cells at their routine maintenance culture density (approximately 4 × 10^5^ cells/mL) were incubated with 1 μg/mL agonistic anti-CD226 antibody (clone NewE1), added directly to the maintenance culture medium without adjusting cell density, previously demonstrated to activate NK-92 cells via DNAM-1 receptor engagement [26], for 24 h at 37 °C in 5% CO_2_. Stimulated cells (sNK-92) were harvested by centrifugation and resuspended in fresh medium. To verify antibody–receptor binding, stimulated NK-92 cells were fixed with 3.5% paraformaldehyde and incubated with a FITC-conjugated goat anti-mouse secondary antibody (Abcam, Cambridge, UK; Cat. No. ab6785) for 2 h at 37 °C. Nuclear counterstaining was performed with 7-AAD (Invitrogen, Thermo Fisher Scientific, Waltham, MA, USA; Cat. No. A1310) for 30 min at 37 °C. Membrane-restricted DNAM-1 receptor localization was confirmed by super-resolution confocal microscopy (SR-CM).

### 4.3. Co-Culture Experimental Setup

Target cells were seeded in 96-well plates at 5 × 10^3^ cells/well and allowed to adhere for 24 h. Target cells were washed twice with PBS, applied uniformly across all wells using a multi-channel pipette; cell retention was confirmed under inverted light microscopy before and after washing. Well-to-well consistency of target-cell number following washing was additionally validated using a separate 96-well plate, seeded and processed identically but outside the experimental design, in which cells were detached and counted after the washing step; this confirmed that cell numbers were not reduced and remained consistent across wells. Subsequently, NK-92 or sNK-92 cells were introduced at target-to-effector (T:E) ratios of 1:1, 1:5, and 1:10 for 4 h at 37 °C in 5% CO_2_ [28]. Co-culture medium consisted of equal volumes (1:1) of each cell type’s respective medium, consistently across all T:E ratios tested; to prevent a dilution-related reduction in IL-2 availability to NK-92 cells resulting from this 1:1 medium mixing, the final IL-2 concentration in the co-culture medium was maintained equivalent to that of standard NK-92 maintenance medium (500 U/mL). Untreated target cells served as the negative control.

### 4.4. CCK-8 Cell Viability Assay

Following 4-h co-culture, NK-92 or sNK-92 effector cells were removed by two washes with PBS, after which CCK-8 reagent (Dojindo Molecular Technologies, Kumamoto, Japan) was added to the remaining adherent target cells and plates were incubated for an additional 2 h at 37 °C. Absorbance was measured at 450 nm using a microplate reader, and cytotoxicity (%) was calculated as: cytotoxicity (%) = 100 − (Absorbance_sample/Absorbance_control × 100), where Absorbance_control represents the absorbance of untreated target cells cultured alone, without NK-92 or sNK-92 co-culture. For each cell line, four experimental groups were evaluated (untreated control and T:E ratios of 1:1, 1:5, and 1:10), each performed with six replicates (*n* = 6).

### 4.5. Immunofluorescence Analysis of Apoptotic Pathway Markers

Target cells seeded at 4 × 10^4^ cells/well on coverslips pre-coated with 0.1 mg/mL poly-L-lysine (Sigma-Aldrich; Cat. No. P4707) were left untreated (control) or co-cultured with NK-92 or sNK-92 cells at the 1:10 T:E ratio for 4 h, selected based on CCK-8 results as the ratio yielding the highest cytotoxic activity against cancer cell lines. Non-adherent cells were removed by three gentle washes with PBS, and adherent target cells were fixed with 3.5% paraformaldehyde (Sigma-Aldrich; Cat. No. 158127). Following fixation, cells were washed three times with PBS and permeabilized with 0.1% Triton X-100 in PBS for 10 min at room temperature. After washing with PBS, non-specific binding sites were blocked with 5% bovine serum albumin (BSA) in PBS for 1 h at room temperature. Cells were then incubated overnight at 4 °C with the following primary antibodies: anti-BAX (Abcam, Cambridge, UK; ab32503; rabbit; 1:250), anti-caspase-3 (CASP3; Abcam; ab184787; rabbit; 1:1000), anti-caspase-8 (CASP8; Elabscience, Houston, TX, USA; E-AB-63511; rabbit; 1:200), and anti-caspase-9 (CASP9; Abcam; ab202068; rabbit; 1:500). Following PBS washes, cells were incubated with FITC-conjugated anti-rabbit secondary antibody (Invitrogen; Cat. No. 656111; 1:2000) for 2 h at 37 °C and counterstained with 7-AAD (Invitrogen; Cat. No. A1310) for 30 min at 37 °C. A secondary antibody-only control (no primary antibody control) was performed in parallel with all immunofluorescence experiments in this study, including the anti-CD226 binding assay described in Section 4.2, to confirm the absence of non-specific secondary antibody binding; no signal was detected in these control samples. Coverslips were mounted with ProLong Gold antifade mounting medium (Thermo Fisher Scientific; Cat. No. P36930) and stored at −20 °C until imaging. All IF images were acquired using a Zeiss Axio Observer inverted fluorescence microscope (Carl Zeiss AG, Oberkochen, Germany) at 40× magnification; scale bar = 50 μm (applies to all panels). Brightness and contrast were adjusted uniformly across entire images to optimize visualization; no region-specific manipulations were performed. Representative merged fluorescence images are presented.

#### Quantitative Analysis of Immunofluorescence Images

Quantitative analysis of the immunofluorescence images was performed using ImageJ software (version 1.54, National Institutes of Health, Bethesda, MD, USA). For each cell line, treatment group, and apoptosis-associated marker, three image fields were analyzed per group. Within each image field, 10 individual target cells were manually delineated as regions of interest (ROIs) on the corresponding green fluorescence channel. Three cell-free regions from each image were selected to determine the mean background fluorescence.

Corrected total cell fluorescence (CTCF) was calculated for each cellular ROI using the following formula:

CTCF = Integrated density − (Cell area × Mean background fluorescence)

The mean CTCF of all analyzed cells within each image was subsequently calculated, resulting in three image-level values for each experimental group. To permit comparison among proteins and cell lines with different baseline fluorescence levels, each image-level CTCF value was divided by the mean CTCF of the corresponding control group. Accordingly, the mean normalized value of each control group was set to 1, and the results were expressed as relative CTCF (fold of control). Individual cellular measurements were treated as subsamples within each image and were not considered independent statistical replicates. Continuous CTCF measurement, rather than the proportion of positive cells, was selected as the primary quantitative outcome, as the markers examined showed a graded increase in signal per cell rather than a binary positive/negative pattern and no biologically validated positivity threshold was available for these markers. Quantification was performed using the original fluorescence images before any adjustments were made for visual presentation.

### 4.6. Statistical Analysis

Data were analyzed using GraphPad Prism (version 10, GraphPad Software, Boston, MA, USA). Normality was assessed by Shapiro–Wilk and Kolmogorov–Smirnov tests. Cytotoxicity data (CCK-8) and quantitative immunofluorescence (CTCF) data were analyzed separately. For CCK-8 assays, inter-group differences relative to the untreated control were evaluated by one-way ANOVA with Tukey’s post hoc test, with results presented as mean ± SD (*n* = 6 technical replicates from a single experimental preparation). Direct comparisons between NK-92 and sNK-92 groups at each T:E ratio were performed separately using an unpaired two-tailed Student’s *t*-test. For the quantitative image analysis, the mean CTCF value obtained from each image field was considered one analytical replicate, with three analyzed image fields per group (*n* = 3); comparisons among the control, NK-92, and sNK-92 groups were performed separately for each cell line and apoptosis-associated marker using ordinary one-way ANOVA followed by Tukey’s multiple-comparisons test. A *p*-value < 0.05 was considered statistically significant in all analyses.

## 5. Conclusions

The present study shows that anti-CD226 antibody-mediated stimulation of NK-92 cells enhances their cytotoxic activity against PC3 and SH-SY5Y cells under the experimental conditions examined. Quantitative immunofluorescence analysis demonstrated pronounced increases in the relative fluorescence intensity of BAX, CASP9, CASP8, and CASP3 in the tumor cell models, particularly following sNK-92 treatment, whereas substantially smaller changes were observed in PNT1A and BJ cells. These findings are consistent with preferential effects on the examined tumor models and with the involvement of apoptosis-associated signaling. However, because these apoptosis-associated marker changes were evaluated primarily by quantitative immunofluorescence and were not independently validated by functional apoptosis or protein-cleavage assays, the findings should be regarded as preliminary. Further studies using independent biological replicates and orthogonal validation methods are required to establish the underlying mechanism and translational relevance.

## Data Availability

The original contributions presented in this study are included in the article. Further inquiries can be directed to the corresponding author.
